# 1-(3-Chloro­phen­yl)-3-(1-*p*-tolyl­imidazolidin-2-yl­idene)urea

**DOI:** 10.1107/S1600536808040701

**Published:** 2008-12-06

**Authors:** Waldemar Wysocki, Dariusz Matosiuk, Zbigniew Karczmarzyk, Sylwia Fidecka, Andrzej Fruziński

**Affiliations:** a Department of Chemistry, University of Podlasie, ul. 3 Maja 54, 08-110 Siedlce, Poland; b Department of Synthesis and Chemical Technology of Pharmaceutical Substances, Medical University, ul. Staszica 6, 20-081 Lublin, Poland; c Department of Pharmacology and Pharmacodynamics, Medical University, ul. Staszica 4, 20-081 Lublin, Poland; d Institute of General and Ecological Chemistry, Technical University, ul. Żwirki 115, 90-924 Łodź, Poland

## Abstract

In the crystal structure of the title compound, C_17_H_17_ClN_4_O, the existence of only one 2-imino–oxo of the five possible *N*-amino–imino/*O*-keto–hydr­oxy tautomers is observed and the dihedral angle between the aromatic rings is 29.78 (11)°. The mol­ecular conformation is stabilized by intra­molecular C—H⋯N, N—H⋯O and C—H⋯O hydrogen bonds, in each case generating a six-membered ring. In the crystal structure, the glide-plane-related mol­ecules are linked into *C*(4) amide chains by inter­molecular N—H⋯O hydrogen bonds, and an inter­molecular C—H⋯O link also occurs.

## Related literature

For general background, synthesis, biological activity and related structures, see: Matosiuk *et al.* (2001 ▶, 2005 ▶); Karczmarzyk *et al.* (2004 ▶); Wysocki *et al.* (2006 ▶). For hydrogen-bond motifs, see: Steiner (2002 ▶); Bernstein *et al.* (1995 ▶).
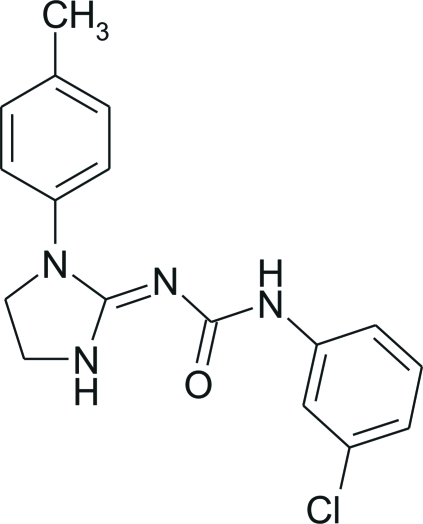


## Experimental

### 

#### Crystal data

C_17_H_17_ClN_4_O
*M* *_r_* = 328.80
Monoclinic, 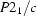

*a* = 11.4506 (8) Å
*b* = 15.5097 (17) Å
*c* = 9.2811 (11) Å
β = 100.506 (8)°
*V* = 1620.7 (3) Å^3^
*Z* = 4
Cu *K*α radiation
μ = 2.17 mm^−1^
*T* = 293 (2) K
0.5 × 0.4 × 0.1 mm

#### Data collection

Kuma KM-4 four-circle diffractometer
Absorption correction: multi-scan (*SORTAV*; Blessing, 1995 ▶) *T*_min_ = 0.398, *T*_max_ = 0.805
4241 measured reflections
3485 independent reflections
2703 reflections with *I* > 2σ(*I*)
*R*_int_ = 0.022
2 standard reflections every 100 reflections intensity decay: 1%

#### Refinement

*R*[*F* ^2^ > 2σ(*F* ^2^)] = 0.055
*wR*(*F* ^2^) = 0.165
*S* = 1.05
3485 reflections
215 parameters
H atoms treated by a mixture of independent and constrained refinement
Δρ_max_ = 0.37 e Å^−3^
Δρ_min_ = −0.25 e Å^−3^

### 

Data collection: *KM4B8* (Gałdecki *et al*., 1996 ▶); cell refinement: *KM4B8*; data reduction: *DATAPROC* (Gałdecki *et al*., 1995 ▶); program(s) used to solve structure: *SHELXS97* (Sheldrick, 2008 ▶); program(s) used to refine structure: *SHELXL97* (Sheldrick, 2008 ▶); molecular graphics: *ORTEP-3* (Farrugia, 1997 ▶); software used to prepare material for publication: *WinGX* (Farrugia, 1999 ▶).

## Supplementary Material

Crystal structure: contains datablocks I, global. DOI: 10.1107/S1600536808040701/hb2873sup1.cif

Structure factors: contains datablocks I. DOI: 10.1107/S1600536808040701/hb2873Isup2.hkl

Additional supplementary materials:  crystallographic information; 3D view; checkCIF report

## Figures and Tables

**Table 1:** Hydrogen-bond geometry (Å, °)

*D*—H⋯*A*	*D*—H	H⋯*A*	*D*⋯*A*	*D*—H⋯*A*
N3—H31⋯O8	0.81 (3)	1.98 (3)	2.611 (2)	134 (3)
C26—H261⋯N6	0.93	2.33	2.919 (3)	121
C32—H321⋯O8	0.93	2.26	2.864 (3)	122
N9—H91⋯O8^i^	0.93 (3)	2.01 (3)	2.927 (2)	171 (2)
C36—H361⋯O8^i^	0.93	2.49	3.266 (2)	141

Symmetry code: (i) 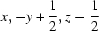.

**Fig. 1.**
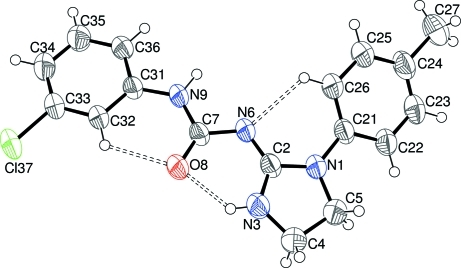
The molecular structure of (I), with 50% probability displacement ellipsoids for non-H atoms.

**Fig. 2.**
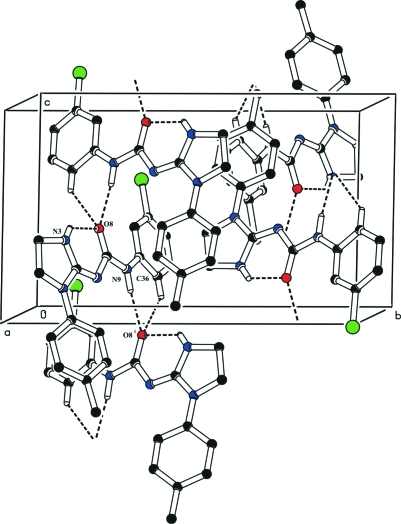
The packing of (I), viewed down the a axis, showing molecules connected by N—H···O and C—H···O hydrogen bonds [symmetry code: (i) x, -y + 1/2, z - 1/2].

## supplementary crystallographic information

### Comment

We report herein the results of the X-ray structure determination of the title compound, (I), as part of a structural study on a tautomeric equilibrium of the chain and fused derivatives of 2-aminoimidazoline with pharmacological activity (Matosiuk *et al.*, 2001, 2005; Karczmarzyk *et al.*, 2004; Wysocki *et al.*, 2006). Investigated compound (I) exhibited central activity associated with agonistic action on opioid mu and delta (MOP and DOP) as well as serotonin (5-HT_2_) receptors. This activity was expressed by its strong antinociceptive, serotonergic and antidepressant action (Matosiuk *et al.*, 2001). In the crystalline state, the compound (I) exists as the N3-amino/N6-imino/O8-keto/N9-amino tautomeric form with the amino and carbonyl groups involved in the strong N3—H31···O8 intramolecular and N9—H91···O8 intermolecular hydrogen bonds with *π*-bond cooperativity (Steiner, 2002) (Table 1 and 2). The geometry (bond lengths, angles and planarity) of the molecule of (I) is very similar to that observed in a previously reported close related structure of 1-(1-phenylimidazolidin-2-ylidene)-3-(4-chlorophenyl)urea (Matosiuk *et al.*, 2001). The *p*-tolyl, 2-iminoimidazoline, urea and chlorophenyl groups are planar to within 0.047 (4), 0.084 (3), 0.006 (2) and 0.006 (2) Å, respectively, and the dihedral angles between mean planes of these groups are in the range of 6.86 (6)–29.88 (6)°. The carbonyl group is in *cis* conformation with the torsion angles C2—N6—C7—O8 and O8—C7—N9—C31 of 1.5 (3) and -7.8 (3)°, respectively. The nearly planar chain-extended conformation of the molecule as a whole is stabilized by the intramolecular C26—H261···N6, N3—H31···O8 and C32—H321···O8 hydrogen bonds (Table 2) leading to the formation of six-ring fused system. In the crystal structure, besides of the N9—H91···O8 intermolecular hydrogen bond linking the glide plane-related molecules into molecular chains along the [001] direction with graph-set motif *C*(4) (Bernstein *et al.*, 1995), the *π*-electron systems of the imidazoline and phenyl rings belonging to inversion-related molecules overlap each other: the shortest intermolecular contact is C2···C22*^j^*=3.292 (3) Å and the angle between the overlapping planes is 16.83°, characteristic of *π*-stacking [symmetry code: (*j*) -*x*, -*y*, -*z*].

### Experimental

The title compound was synthesized from respective aniline *via N*-aryloethylenediamine, 1-aryl-2-aminoimidazoline-2 and final condensation with aromatic isocyanate according to the method of Matosiuk *et al.*, (2001).  Colourless prisms of (I) were grown by slow evaporation of an ethanol solution.

### Refinement

N-bound H atoms were located by difference Fourier synthesis and refined freely. The remaining H atoms were positioned geometrically and treated as riding on their C atoms, with C—H distances of 0.93 (aromatic), 0.97 (CH_2_) and 0.96 Å (CH_3_). All H atoms were assigned *U*_iso_(H) values of 1.5*U*_eq_(N,*C*).

### Crystal data

**Table:**

C_17_H_17_ClN_4_O	*F*(000) = 688
*M**_r_* = 328.80	*D*_x_ = 1.347 Mg m^−^^3^
Monoclinic, *P*2_1_/*c*	Cu *K*α radiation, λ = 1.54178 Å
Hall symbol: -P 2ybc	Cell parameters from 25 reflections
*a* = 11.4506 (8) Å	θ = 20.4–37.9°
*b* = 15.5097 (17) Å	µ = 2.17 mm^−^^1^
*c* = 9.2811 (11) Å	*T* = 293 K
β = 100.506 (8)°	Prism, colourless
*V* = 1620.7 (3) Å^3^	0.5 × 0.4 × 0.1 mm
*Z* = 4	

### Data collection

**Table:**

Kuma KM-4 four-circle diffractometer	*R*_int_ = 0.022
graphite	θ_max_ = 80.3°, θ_min_ = 3.9°
ω–2θ scans	*h* = −14→1
Absorption correction: multi-scan (*SORTAV*; Blessing, 1995)	*k* = −19→1
*T*_min_ = 0.398, *T*_max_ = 0.805	*l* = −11→11
4241 measured reflections	2 standard reflections every 100 reflections
3485 independent reflections	intensity decay: 1%
2703 reflections with *I* > 2σ(*I*)	

### Refinement

**Table:**

Refinement on *F*^2^	Secondary atom site location: difference Fourier map
Least-squares matrix: full	Hydrogen site location: mixed
*R*[*F*^2^ > 2σ(*F*^2^)] = 0.055	H atoms treated by a mixture of independent and constrained refinement
*wR*(*F*^2^) = 0.165	*w* = 1/[σ^2^(*F*_o_^2^) + (0.1001*P*)^2^ + 0.2753*P*] where *P* = (*F*_o_^2^ + 2*F*_c_^2^)/3
*S* = 1.05	(Δ/σ)_max_ < 0.001
3485 reflections	Δρ_max_ = 0.37 e Å^−^^3^
215 parameters	Δρ_min_ = −0.25 e Å^−^^3^
0 restraints	Extinction correction: *SHELXL97* (Sheldrick, 2008)
Primary atom site location: structure-invariant direct methods	Extinction coefficient: 0.0059 (8)

### Special details

**Table:**

Geometry. All e.s.d.'s (except the e.s.d. in the dihedral angle between two l.s. planes) are estimated using the full covariance matrix. The cell e.s.d.'s are taken into account individually in the estimation of e.s.d.'s in distances, angles and torsion angles; correlations between e.s.d.'s in cell parameters are only used when they are defined by crystal symmetry. An approximate (isotropic) treatment of cell e.s.d.'s is used for estimating e.s.d.'s involving l.s. planes. Weighted least-squares planes through the starred atoms (Nardelli, Musatti, Domiano & Andreetti Ric.Sci.(1965),15(II—A),807). Equation of the plane: m1**X*+m2*Y+m3**Z*=dPlane 1 m1 = 0.90393(0.00040) m2 = 0.30333(0.00097) m3 = -0.30149(0.00070) D = -1.33176(0.00722) Atom d s d/s (d/s)**2 C21 * -0.0098 0.0019 - 5.243 27.491 C22 * -0.0036 0.0021 - 1.701 2.893 C23 * 0.0143 0.0022 6.386 40.780 C24 * 0.0139 0.0024 5.729 32.824 C25 * 0.0142 0.0029 4.973 24.734 C26 * 0.0023 0.0026 0.875 0.766 C27 * -0.0475 0.0036 - 13.219 174.733 ============ Sum((d/s)**2) for starred atoms 304.221 Chi-squared at 95% for 4 degrees of freedom: 9.49 The group of atoms deviates significantly from planarityPlane 2 m1 = 0.80521(0.00072) m2 = 0.56672(0.00100) m3 = -0.17457(0.00103) D = 1.23989(0.01043) Atom d s d/s (d/s)**2 N1 * -0.0082 0.0017 - 4.965 24.648 C2 * 0.0362 0.0018 20.251 410.085 N3 * -0.0538 0.0019 - 28.155 792.719 C4 * 0.0837 0.0027 31.025 962.555 C5 * -0.0338 0.0026 - 13.108 171.815 ============ Sum((d/s)**2) for starred atoms 2361.821 Chi-squared at 95% for 2 degrees of freedom: 5.99 The group of atoms deviates significantly from planarityPlane 3 m1 = 0.71121(0.00067) m2 = 0.67408(0.00074) m3 = -0.19949(0.00094) D = 1.85835(0.00799) Atom d s d/s (d/s)**2 N6 * 0.0013 0.0015 0.821 0.673 C7 * -0.0058 0.0019 - 3.151 9.926 N9 * 0.0014 0.0017 0.843 0.710 O8 * 0.0015 0.0015 0.999 0.997 ============ Sum((d/s)**2) for starred atoms 12.306 Chi-squared at 95% for 1 degrees of freedom: 3.84 The group of atoms deviates significantly from planarityPlane 4 m1 = 0.62657(0.00064) m2 = 0.73566(0.00060) m3 = -0.25731(0.00040) D = 1.62379(0.00373) Atom d s d/s (d/s)**2 C31 * 0.0038 0.0018 2.177 4.737 C32 * -0.0021 0.0021 - 0.989 0.978 C33 * 0.0061 0.0024 2.568 6.596 C34 * 0.0044 0.0025 1.711 2.926 C35 * 0.0004 0.0024 0.180 0.032 C36 * -0.0065 0.0020 - 3.250 10.562 Cl37 * -0.0008 0.0009 - 0.883 0.780 ============ Sum((d/s)**2) for starred atoms 26.612 Chi-squared at 95% for 4 degrees of freedom: 9.49 The group of atoms deviates significantly from planarityDihedral angles formed by LSQ-planes Plane - plane angle (s.u.) angle (s.u.) 1 2 17.75 (0.07) 162.25 (0.07) 1 3 24.84 (0.07) 155.16 (0.07) 1 4 29.88 (0.06) 150.12 (0.06) 2 3 8.31 (0.06) 171.69 (0.06) 2 4 14.91 (0.06) 165.09 (0.06) 3 4 6.86 (0.06) 173.14 (0.06)
Refinement. Refinement of *F*^2^ against ALL reflections. The weighted *R*-factor *wR* and goodness of fit *S* are based on *F*^2^, conventional *R*-factors *R* are based on *F*, with *F* set to zero for negative *F*^2^. The threshold expression of *F*^2^ > σ(*F*^2^) is used only for calculating *R*-factors(gt) *etc*. and is not relevant to the choice of reflections for refinement. *R*-factors based on *F*^2^ are statistically about twice as large as those based on *F*, and *R*- factors based on ALL data will be even larger.

### Fractional atomic coordinates and isotropic or equivalent isotropic displacement parameters (Å^2^)

**Table:**

	*x*	*y*	*z*	*U*_iso_*/*U*_eq_	
N1	−0.09936 (14)	0.43729 (11)	0.57376 (17)	0.0563 (4)	
C2	−0.01993 (15)	0.39274 (12)	0.67403 (19)	0.0492 (4)	
N3	−0.01904 (16)	0.42651 (13)	0.80768 (19)	0.0627 (5)	
H31	0.034 (3)	0.408 (2)	0.870 (3)	0.094*	
C4	−0.0828 (2)	0.50708 (18)	0.8019 (2)	0.0739 (6)	
H41	−0.0287	0.5554	0.8233	0.111*	
H42	−0.1376	0.5073	0.8702	0.111*	
C5	−0.1490 (2)	0.50998 (17)	0.6438 (2)	0.0709 (6)	
H51	−0.2338	0.5029	0.639	0.106*	
H52	−0.1348	0.5641	0.5974	0.106*	
N6	0.04359 (13)	0.32839 (10)	0.63777 (16)	0.0493 (4)	
C7	0.12813 (16)	0.29293 (12)	0.74519 (18)	0.0476 (4)	
O8	0.15118 (13)	0.31364 (10)	0.87643 (14)	0.0618 (4)	
N9	0.18900 (14)	0.22979 (11)	0.68843 (16)	0.0527 (4)	
H91	0.170 (2)	0.2200 (17)	0.588 (3)	0.079*	
C21	−0.14323 (16)	0.41676 (13)	0.4251 (2)	0.0535 (4)	
C22	−0.20757 (18)	0.47920 (14)	0.3367 (2)	0.0588 (5)	
H221	−0.2174	0.5337	0.3746	0.088*	
C23	−0.25711 (19)	0.46083 (16)	0.1925 (2)	0.0647 (5)	
H231	−0.2992	0.5037	0.135	0.097*	
C24	−0.2459 (2)	0.38109 (17)	0.1321 (2)	0.0699 (6)	
C25	−0.1811 (3)	0.31957 (18)	0.2211 (3)	0.0819 (8)	
H251	−0.1716	0.2652	0.1825	0.123*	
C26	−0.1300 (2)	0.33600 (16)	0.3655 (2)	0.0717 (6)	
H261	−0.087	0.2932	0.4223	0.108*	
C27	−0.3058 (3)	0.3593 (2)	−0.0224 (3)	0.0987 (10)	
H271	−0.3846	0.3385	−0.0216	0.148*	
H272	−0.261	0.3157	−0.0613	0.148*	
H273	−0.31	0.4101	−0.0823	0.148*	
C31	0.29140 (16)	0.18486 (11)	0.75469 (18)	0.0475 (4)	
C32	0.34463 (19)	0.19435 (13)	0.9002 (2)	0.0597 (5)	
H321	0.3127	0.2318	0.9611	0.09*	
C33	0.4462 (2)	0.14726 (15)	0.9541 (2)	0.0665 (6)	
C34	0.4955 (2)	0.09095 (17)	0.8697 (3)	0.0713 (6)	
H341	0.5634	0.0598	0.9083	0.107*	
C35	0.4410 (2)	0.08155 (16)	0.7248 (3)	0.0674 (6)	
H351	0.4732	0.0436	0.665	0.101*	
C36	0.33986 (17)	0.12737 (13)	0.6673 (2)	0.0536 (4)	
H361	0.3042	0.1197	0.5698	0.08*	
Cl37	0.51115 (8)	0.16036 (6)	1.13661 (8)	0.1143 (4)	

### Atomic displacement parameters (Å^2^)

**Table:**

	*U*^11^	*U*^22^	*U*^33^	*U*^12^	*U*^13^	*U*^23^
N1	0.0552 (8)	0.0643 (9)	0.0447 (8)	0.0130 (7)	−0.0037 (6)	−0.0031 (7)
C2	0.0462 (8)	0.0592 (10)	0.0393 (8)	0.0018 (7)	0.0004 (6)	0.0012 (7)
N3	0.0623 (10)	0.0803 (12)	0.0424 (8)	0.0166 (9)	0.0014 (7)	−0.0065 (8)
C4	0.0757 (14)	0.0855 (16)	0.0569 (12)	0.0241 (12)	0.0029 (10)	−0.0130 (11)
C5	0.0685 (12)	0.0811 (15)	0.0590 (12)	0.0232 (11)	0.0005 (10)	−0.0102 (11)
N6	0.0500 (8)	0.0569 (8)	0.0367 (7)	0.0070 (6)	−0.0037 (6)	0.0010 (6)
C7	0.0512 (9)	0.0543 (9)	0.0337 (7)	0.0017 (7)	−0.0021 (6)	0.0031 (7)
O8	0.0720 (9)	0.0738 (9)	0.0345 (6)	0.0170 (7)	−0.0037 (6)	−0.0026 (6)
N9	0.0583 (8)	0.0614 (9)	0.0329 (7)	0.0117 (7)	−0.0063 (6)	−0.0008 (6)
C21	0.0470 (9)	0.0655 (11)	0.0436 (9)	0.0047 (8)	−0.0029 (7)	0.0019 (8)
C22	0.0542 (10)	0.0605 (11)	0.0569 (11)	0.0009 (8)	−0.0025 (8)	0.0087 (9)
C23	0.0574 (11)	0.0751 (13)	0.0553 (11)	0.0018 (10)	−0.0065 (8)	0.0169 (10)
C24	0.0666 (12)	0.0888 (16)	0.0472 (10)	0.0055 (11)	−0.0084 (9)	0.0040 (10)
C25	0.0937 (17)	0.0854 (16)	0.0540 (12)	0.0259 (14)	−0.0203 (12)	−0.0114 (11)
C26	0.0812 (14)	0.0739 (14)	0.0497 (11)	0.0231 (11)	−0.0155 (10)	−0.0047 (9)
C27	0.112 (2)	0.115 (2)	0.0541 (13)	0.0148 (18)	−0.0255 (14)	−0.0012 (14)
C31	0.0506 (9)	0.0489 (9)	0.0394 (8)	0.0016 (7)	−0.0011 (7)	0.0032 (7)
C32	0.0669 (12)	0.0619 (11)	0.0431 (9)	0.0141 (9)	−0.0092 (8)	−0.0031 (8)
C33	0.0681 (12)	0.0695 (13)	0.0521 (11)	0.0138 (10)	−0.0147 (9)	−0.0001 (9)
C34	0.0628 (12)	0.0794 (14)	0.0648 (13)	0.0215 (11)	−0.0067 (10)	0.0016 (11)
C35	0.0631 (12)	0.0762 (14)	0.0602 (12)	0.0149 (10)	0.0044 (9)	−0.0041 (10)
C36	0.0555 (10)	0.0597 (10)	0.0429 (9)	0.0028 (8)	0.0024 (7)	−0.0005 (8)
Cl37	0.1317 (7)	0.1242 (7)	0.0627 (4)	0.0537 (5)	−0.0466 (4)	−0.0179 (4)

### Geometric parameters (Å, °)

**Table:**

N1—C2	1.365 (2)	C23—C24	1.374 (4)
N1—C21	1.416 (2)	C23—H231	0.93
N1—C5	1.467 (3)	C24—C25	1.386 (3)
C2—N6	1.314 (2)	C24—C27	1.510 (3)
C2—N3	1.345 (2)	C25—C26	1.385 (3)
N3—C4	1.443 (3)	C25—H251	0.93
N3—H31	0.81 (3)	C26—H261	0.93
C4—C5	1.524 (3)	C27—H271	0.96
C4—H41	0.97	C27—H272	0.96
C4—H42	0.97	C27—H273	0.96
C5—H51	0.97	C31—C32	1.385 (2)
C5—H52	0.97	C31—C36	1.388 (3)
N6—C7	1.372 (2)	C32—C33	1.387 (3)
C7—O8	1.241 (2)	C32—H321	0.93
C7—N9	1.363 (2)	C33—C34	1.362 (3)
N9—C31	1.406 (2)	C33—Cl37	1.734 (2)
N9—H91	0.93 (3)	C34—C35	1.383 (3)
C21—C26	1.388 (3)	C34—H341	0.93
C21—C22	1.390 (3)	C35—C36	1.381 (3)
C22—C23	1.384 (3)	C35—H351	0.93
C22—H221	0.93	C36—H361	0.93
			
C2—N1—C21	128.66 (16)	C24—C23—H231	119.1
C2—N1—C5	110.55 (16)	C22—C23—H231	119.1
C21—N1—C5	120.49 (15)	C23—C24—C25	117.1 (2)
N6—C2—N3	128.35 (17)	C23—C24—C27	121.9 (2)
N6—C2—N1	122.75 (16)	C25—C24—C27	120.9 (2)
N3—C2—N1	108.89 (17)	C26—C25—C24	122.4 (2)
C2—N3—C4	112.58 (17)	C26—C25—H251	118.8
C2—N3—H31	113 (2)	C24—C25—H251	118.8
C4—N3—H31	130 (2)	C25—C26—C21	119.6 (2)
N3—C4—C5	102.76 (17)	C25—C26—H261	120.2
N3—C4—H41	111.2	C21—C26—H261	120.2
C5—C4—H41	111.2	C24—C27—H271	109.5
N3—C4—H42	111.2	C24—C27—H272	109.5
C5—C4—H42	111.2	H271—C27—H272	109.5
H41—C4—H42	109.1	C24—C27—H273	109.5
N1—C5—C4	103.82 (17)	H271—C27—H273	109.5
N1—C5—H51	111	H272—C27—H273	109.5
C4—C5—H51	111	C32—C31—C36	119.13 (16)
N1—C5—H52	111	C32—C31—N9	123.91 (17)
C4—C5—H52	111	C36—C31—N9	116.96 (15)
H51—C5—H52	109	C31—C32—C33	119.11 (19)
C2—N6—C7	117.94 (15)	C31—C32—H321	120.4
O8—C7—N9	122.24 (16)	C33—C32—H321	120.4
O8—C7—N6	127.25 (17)	C34—C33—C32	122.6 (2)
N9—C7—N6	110.50 (14)	C34—C33—Cl37	119.23 (16)
C7—N9—C31	129.19 (14)	C32—C33—Cl37	118.18 (17)
C7—N9—H91	117.4 (17)	C33—C34—C35	117.79 (19)
C31—N9—H91	112.5 (17)	C33—C34—H341	121.1
C26—C21—C22	118.55 (18)	C35—C34—H341	121.1
C26—C21—N1	123.07 (17)	C36—C35—C34	121.3 (2)
C22—C21—N1	118.30 (18)	C36—C35—H351	119.4
C23—C22—C21	120.4 (2)	C34—C35—H351	119.4
C23—C22—H221	119.8	C35—C36—C31	120.09 (17)
C21—C22—H221	119.8	C35—C36—H361	120
C24—C23—C22	121.85 (19)	C31—C36—H361	120
			
C21—N1—C2—N6	11.5 (3)	N1—C21—C22—C23	−176.89 (19)
C5—N1—C2—N6	−175.0 (2)	C21—C22—C23—C24	0.6 (3)
C21—N1—C2—N3	−169.24 (19)	C22—C23—C24—C25	−0.9 (4)
C5—N1—C2—N3	4.3 (2)	C22—C23—C24—C27	176.4 (3)
N6—C2—N3—C4	168.4 (2)	C23—C24—C25—C26	0.6 (4)
N1—C2—N3—C4	−10.8 (3)	C27—C24—C25—C26	−176.7 (3)
C2—N3—C4—C5	12.3 (3)	C24—C25—C26—C21	0.0 (5)
C2—N1—C5—C4	3.2 (3)	C22—C21—C26—C25	−0.3 (4)
C21—N1—C5—C4	177.4 (2)	N1—C21—C26—C25	176.5 (2)
N3—C4—C5—N1	−8.8 (3)	C7—N9—C31—C32	5.0 (3)
N3—C2—N6—C7	−4.7 (3)	C7—N9—C31—C36	−175.62 (19)
N1—C2—N6—C7	174.45 (17)	C36—C31—C32—C33	1.1 (3)
C2—N6—C7—O8	1.5 (3)	N9—C31—C32—C33	−179.5 (2)
C2—N6—C7—N9	−177.46 (17)	C31—C32—C33—C34	−0.8 (4)
O8—C7—N9—C31	−7.8 (3)	C31—C32—C33—Cl37	179.97 (17)
N6—C7—N9—C31	171.18 (18)	C32—C33—C34—C35	0.3 (4)
C2—N1—C21—C26	14.4 (3)	Cl37—C33—C34—C35	179.6 (2)
C5—N1—C21—C26	−158.6 (2)	C33—C34—C35—C36	−0.2 (4)
C2—N1—C21—C22	−168.8 (2)	C34—C35—C36—C31	0.6 (4)
C5—N1—C21—C22	18.2 (3)	C32—C31—C36—C35	−1.1 (3)
C26—C21—C22—C23	0.0 (3)	N9—C31—C36—C35	179.5 (2)

### Hydrogen-bond geometry (Å, °)

**Table:**

*D*—H···*A*	*D*—H	H···*A*	*D*···*A*	*D*—H···*A*
N3—H31···O8	0.81 (3)	1.98 (3)	2.611 (2)	134 (3)
C26—H261···N6	0.93	2.33	2.919 (3)	121
C32—H321···O8	0.93	2.26	2.864 (3)	122
N9—H91···O8^i^	0.93 (3)	2.01 (3)	2.927 (2)	171 (2)
C36—H361···O8^i^	0.93	2.49	3.266 (2)	141

Symmetry codes: (i) *x*, −*y*+1/2, *z*−1/2.
